# Supplementary material for: Identification of photoperiod-induced specific miRNAs in the adrenal glands of Sunite sheep (Ovis aries)
Source: Front Vet Sci. 2022 Jul 22;9:888207. doi: 10.3389/fvets.2022.888207 (PMC9354845; doi:10.3389/fvets.2022.888207)
Supplement: Supplementary file 1 [file Data_Sheet_1.ZIP › Supplementary file/miRDeep2 file.zip!/miRDeep2 file/Know_Structure/SPLP42A1/oar-mir-99a.pdf]

miRBase precursor : oar-mir-99a
Total read count : 642726
oar-miR-99a read count : 642187
remaining reads : 539

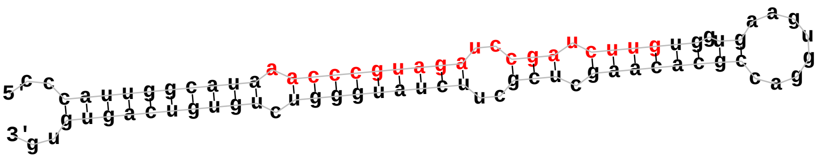

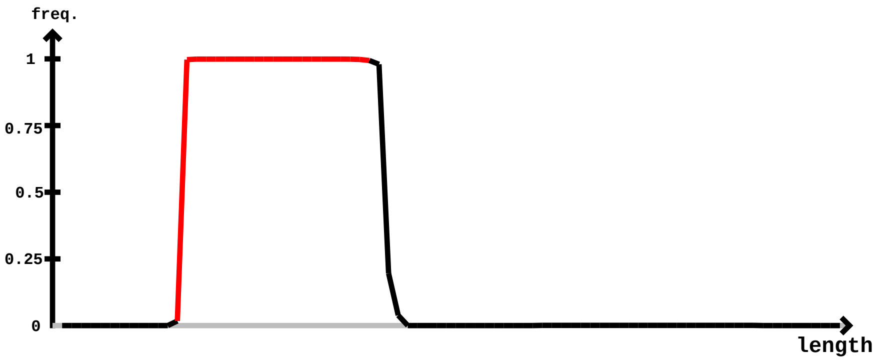

oar-miR-99a

5'- cccauuggcauaaacccguagauccgaucuuguggugaaguggaccgcacaagcucgcuucuaugggucugugucagugug -3'	exp		
..((((((((((.(((((((((..(((.((((((.((........)))))))).)))..))))))))).))))))))))..	reads	mm	sample
...........aaacccguagauccgauc..	5	0	seq
...........aaacccguagauccgaucu...	10	0	seq
...........aaacccguagauccgaucuu..	45	0	seq
...........aaacccguagauccgaucuug...	131	0	seq
...........aaacccguagauccgaucuugu..	10556	0	seq
...........aaacccguagauccgaAcuugu..	8	1	seq
...........aaacccguagauccgaucuugug...	261	0	seq
...........aaacccguagauccgaucuugugg..	28	0	seq
............aacccguagauccgaAc..	3	1	seq
............aacccguagauccgauc..	89	0	seq
............aacccguagauccgaAcu...	46	1	seq
............aacccguagauccgaucu...	705	0	seq
............aacccguagauccgaucuu..	2390	0	seq
............aacccguagauccgaAcuu..	93	1	seq
............aacccguagauccgaucuug...	8143	0	seq
............aacccguagauccgaAcuug...	737	1	seq
............aacccguagauccgaucuugu..	480846	0	seq
............aacccguagauccgaAcuugu..	11856	1	seq
............aacccguagauccgaAcuugug...	17862	1	seq
............aacccguagauccgaucuugug...	82481	0	seq
............aacccguagauccgaucuugugg..	24092	0	seq
............aacccguagauccgaAcuugugg..	538	1	seq
............aacccguagauccgaucuuguggu...	23	0	seq
............aacccguagauccgaAcuuguggu...	1	1	seq
.............acccguagauccgaucu...	1	0	seq
.............acccguagauccgaucuu..	1	0	seq
.............acccguagauccgaAcuu..	1	1	seq
.............acccguagauccgaAcuug...	6	1	seq
.............acccguagauccgaucuug...	7	0	seq
.............acccguagauccgaAcuugu..	126	1	seq
.............acccguagauccgaucuugu..	539	0	seq
.............acccguagauccgaucuugug...	92	0	seq
.............acccguagauccgaAcuugug...	340	1	seq
.............acccguagauccgaAcuugugg..	19	1	seq
.............acccguagauccgaucuugugg..	35	0	seq

oar-miR-99a

```
cccauuggcauaaacccguagauccgaucuuguggugaaguggaccgcacaagcucgcuucuaugggucugugucagugug
..............cccguagauccgaucuugu................................................    9      0     seq
...............ccguagauccgaucuugu................................................    28     0     seq
...............ccguagauccgaAcuugu................................................    1      1     seq
...............ccguagauccgaAcuugug...............................................    1      1     seq
...............ccguagauccgaucuugug...............................................    3      0     seq
...............ccguagauccgaucuugugg..............................................    5      0     seq
................cguagauccgaucuugu................................................    12     0     seq
................cguagauccgaucuugug...............................................    8      0     seq
................cguagauccgaucuugugg..............................................    3      0     seq
.................guagauccgaucuugugg..............................................    1      0     seq
.................................................caagcucgcuucuaugggu.............    1      0     seq
.................................................caagcucgcuucuaugggucu...........    5      0     seq
.................................................caagcucgcuucuaugggucug..........    7      0     seq
.................................................caagcucgcuucuaugggucugu.........    511    0     seq
.................................................caagcucgcuucuaugggucugug........    3      0     seq
..................................................aagcucgcuucuaugggucugu.........    12     0     seq
```
